# The Interaction of Compliance and Activation on the Force-Length Operating Range and Force Generating Capacity of Skeletal Muscle: A Computational Study using a Guinea Fowl Musculoskeletal Model

**DOI:** 10.1093/iob/obz022

**Published:** 2019-09-03

**Authors:** S M Cox, K L Easton, M Cromie Lear, R L Marsh, S L Delp, J Rubenson

**Affiliations:** 1 Biomechanics Laboratory, Kinesiology Department, The Pennsylvania State University, University Park, PA 16802, USA; 2 School of Human Sciences, The University of Western Australia, Perth, WA 6009, Australia; 3 Department of Mechanical Engineering, Stanford University, Stanford, CA 94305, USA; 4 Department of Biology, Northeastern University, Boston, MA 02115, USA; 5 Department of Ecology and Evolutionary Biology, Brown University, Providence, RI 02912, USA; 6 Departments of Bioengineering and Orthopedic Surgery, Stanford University, Stanford, CA 94305, USA

## Abstract

A muscle’s performance is influenced by where it operates on its force–length (F–L) curve. Here we explore how activation and tendon compliance interact to influence muscle operating lengths and force-generating capacity. To study this, we built a musculoskeletal model of the lower limb of the guinea fowl and simulated the F–L operating range during fixed-end fixed-posture contractions for 39 actuators under thousands of combinations of activation and posture using three different muscle models: Muscles with non-compliant tendons, muscles with compliant tendons but no activation-dependent shift in optimal fiber length (L0), and muscles with both compliant tendons and activation-dependent shifts in L0. We found that activation-dependent effects altered muscle fiber lengths up to 40% and increased or decreased force capacity by up to 50% during fixed-end contractions. Typically, activation-compliance effects reduce muscle force and are dominated by the effects of tendon compliance at high activations. At low activation, however, activation-dependent shifts in L0 are equally important and can result in relative force changes for low compliance muscles of up to 60%. There are regions of the F–L curve in which muscles are most sensitive to compliance and there are troughs of influence where these factors have little effect. These regions are hard to predict, though, because the magnitude and location of these areas of high and low sensitivity shift with compliance level. In this study we provide a map for when these effects will meaningfully influence force capacity and an example of their contributions to force production during a static task, namely standing.

## Synopsis

A Interação de Conformidade e Ativação na Faixa de Operação Força-Comprimento e Capacidade de Geração de Força do Músculo Esquelético: Um Estudo Computacional Usando um Modelo Musculoesquelético de Galinhas-D’angola O desempenho muscular é influenciado por onde ele opera na sua curva de força-comprimento. Aqui, exploramos como a ativação e a conformidade do tendão interagem para influenciar os comprimentos musculares e a capacidade de geração de força. Para estudar isso, construímos um modelo musculoesquelético do membro inferior da galinha-d’angola e simulamos a faixa de operação força-comprimento durante contrações fixas de postura e extremidade para 39 atuadores sob milhares de combinações de ativação e postura usando três modelos musculares diferentes: músculos com tendões não-complacentes, músculos com tendões complacentes, mas sem desvio dependente de ativação no comprimento ideal de fibra (L0), e músculos com tendões complacentes e desvios dependentes de ativação em L0. Descobrimos que os efeitos dependentes da ativação alteraram os comprimentos da fibra muscular em até 40% e aumentaram ou diminuíram a capacidade de força em até 50% durante as contrações de extremidade fixas. Normalmente, os efeitos de ativação e conformidade reduzem a força muscular e são dominados pelos efeitos de complacência do tendão em altas ativações. Em baixa ativação, no entanto, desvios dependentes de ativação em L0 são igualmente importantes e podem resultar em mudanças de força relativas de até 60% para músculos de baixa complacência. Existem regiões da curva de força-comprimento em que os músculos são mais sensíveis à complacência e há baixas de influência onde esses fatores têm pouco efeito. Essas regiões são difíceis de prever porque a magnitude e a localização dessas áreas de alta e baixa sensibilidade mudam com o nível de conformidade. Neste estudo, fornecemos um mapa para quando esses efeitos influenciarão significativamente a capacidade de força e um exemplo de suas contribuições para a produção de forças durante uma tarefa estática, ou seja, em pé. Translated to Portuguese by G. Sobral (gabisobral@gmail.com)

## Introduction

Where muscles operate on their force–length (F–L) relationship has important implications for muscle and locomotor performance. Most tangibly, muscle length affects muscle force and, therefore, joint torque capacity (Blix 1894; Ramsey and Street 1941; Gordon et al. 1966; Rack and Westbury 1969; Huxley and Simmons 1971; ter Keurs et al. 1978; Rassier et al. 1999; Morgan et al. 2002; Arnold et al. 2013). One relatively unexplored, but potentially significant, factor influencing muscle operating lengths is muscle–tendon compliance. Zajac (1989) initially suggested that even in “isometric” (fixed-end) contractions, muscle fibers will shorten due to the stretch of the tendon, an effect exaggerated at higher activation levels due to greater forces. The result is that the operating ranges for muscles with compliant tendons shift to the left on the F–L curve with increasing activation. This theoretical prediction has been experimentally confirmed in several studies (Lakatta and Jewell 1977; Fukunaga et al. 1997; Hawkins and Bey 1997; MacIntosh and MacNaughton 2005; Lemos et al. 2008; Azizi and Roberts 2010; Arnold and Delp 2011; Sugisaki et al. 2011; Rubenson et al. 2012; Holt and Azizi 2016; Mayfield et al. 2016). More recently, it has been shown that tendon compliance is an important factor affecting muscle F–L operating ranges during human gait (Arnold and Delp 2011). Yet, despite many studies demonstrating this effect for individual muscles or during specific tasks, we still do not have a broad understanding of when tendon compliance has a meaningful influence on muscle operating length and function. For instance: Is there a threshold of muscle–tendon compliance where the effect becomes functionally relevant? Does compliance have a consistent influence on the F–L operating range in all conditions (i.e., across different postures and muscle lengths) or are there conditions when it is more influential?

Complicating these questions is the observation that, even in the absence of muscle–tendon compliance, activation can alter optimal muscle lengths. While the exact mechanisms remain unclear, a rightward shift of the plateau region of the F–L curve occurs with decreasing activation (Rack and Westbury 1969; Roszek et al. 1994; Holt and Azizi 2014) such that the optimal muscle length for force production increases. Although an interaction between activation and compliance on muscle operating lengths has been demonstrated (Lieber and Brown 1992; Lemos et al. 2008; Arnold and Delp 2011; Arnold et al. 2013; Rubenson et al. 2013), no studies have systematically explored the simultaneous influence of activation-dependent optimal fiber length and tendon compliance, and its subsequent effect on F–L operating ranges. Nor have the relative contributions of compliance and activation-dependent shifts of the F–L curve been quantified. Therefore, we do not know under what conditions we can safely ignore these complicating factors in interpreting and predicting how muscle force varies with length across any species.

**Fig. 1:**
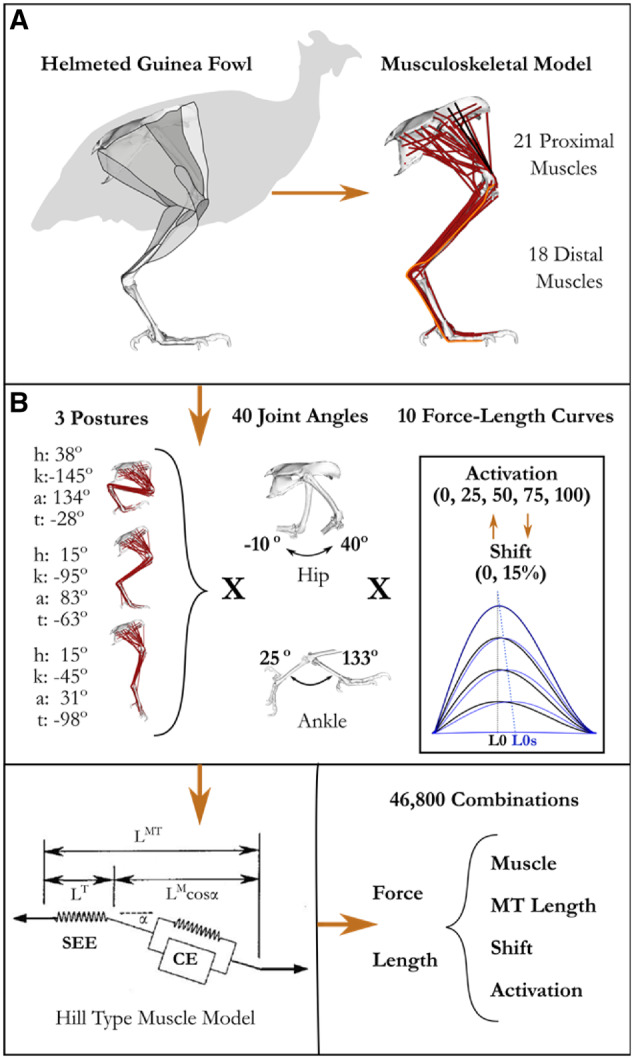
Methodological approach. **A**) Musculoskeletal model was developed in SIMM and implemented in OpenSim (see Supplementary Material). **B**) Since several muscles cross multiple joints, we varied starting MTU length by changing the posture at the hip (h), knee (k), ankle (a), and the tarso metaphalangeal (t) joints and then swept the hip and ankle through a series of additional joint deviations. At each joint configuration, we simulated fixed-end muscle contraction at five levels of activation. At each level of activation, we implemented three different muscle models 1) muscles with a non-compliant tendon, 2) muscles with a compliant tendons, and 3) muscles with compliant tendons and a 15% activation dependent shift of the optimal fiber length. For each joint configuration and muscle model, we extracted the equilibrium muscle length and force capacity of each muscle during the fixed end contraction. With this data we evaluated how muscle length and force capacity changed with muscle activation level, passive muscle length, muscle–tendon unit compliance, and activation-dependent shifts of the F–L curve.

The purpose of this study is to undertake the first comprehensive assessment of how tendon elasticity and activation-dependent shifts in optimal fiber lengths combine to influence F–L operating ranges. To this end, we integrated a non-human animal model and computational approach, the combination of which is well suited to illuminate these relationships. We developed a computational musculoskeletal model of the guinea fowl pelvic-limb (Fig. 1A), a popular avian bipedal model for biomechanical studies (Fedak et al. 1974, 1982; Gatesy 1999; Allen et al. 2017; Ellerby et al. 2005; Daley et al. 2006; Marsh et al. 2006; Rubenson and Marsh 2009; Ellerby and Marsh 2010; Daley and Biewener 2011; Gordon et al. 2015). An animal computational model has the advantage of including highly detailed measurements of musculoskeletal parameters, many of which are typically not included in current human models (see Supplementary Material). Additionally, in this animal model the scope of muscle–tendon compliance (Table 1) is twice that of humans (Arnold and Delp 2011), amplifying the effects. Using a computational approach allowed us to iterate many more combinations of activation and posture than would be possible experimentally. We simulated the F–L operating range for 39 actuators (Table 1) under thousands of combinations of activation and posture to distill the influence of tendon elasticity and activation on F–L operating range (Fig. 1). This computational approach enabled us to extrapolate overall patterns and to systematically tease out the contributions of individual factors. For example, this method allowed us to discriminate the role of the initial passive (pre-activation) lengths of muscles, activation-dependent optimal fiber lengths, and tendon compliance on muscle operating length.

**Table 1:** Tendon slack length (TSL), optimal fiber length (L0), and their ratio listed for each muscle used for analysis

Muscle	TSL (m)	L0 (m)	T/M ratio	Muscle	TSL (m)	L0 (m)	T/M ratio
Hip flexion/extension muscles				Ankle flexion/extension muscles			
CFP	0.027	0.037	0.71	DFii_d	0.182	0.011	16.55
FCLP_c	0	0.120	0.00	DFii_dx	0.185	0.015	12.33
FCLP_p	0.078	0.054	1.46	DFiii_dx	0.196	0.016	12.33
IC_cr	0	0.086	0.00	DFiii_d	0.195	0.013	14.77
IC_cd	0	0.085	0.00	EDL_iii	0.193	0.012	15.69
ILPO_cr	0.064	0.033	1.93	FHL_iii	0.239	0.020	11.75
ILPO_m	0.017	0.083	0.21	FL_l	0.084	0.049	1.73
ILPO_cd	0	0.111	0.00	FL_p	0.081	0.049	1.67
ILPR_cr	0.044	0.041	1.09	IG	0.075	0.072	1.05
ILPR_cd	0.057	0.026	2.19	LG	0.102	0.047	2.17
ISF_v	0.039	0.014	2.76	MG_l	0.105	0.041	2.57
ISF_d	0.039	0.014	2.76	MG_c	0.118	0.037	3.17
ITC_d	0.019	0.023	0.83	MG_m	0.120	0.028	4.23
ITC_v	0.012	0.028	0.42	TC_f	0.119	0.029	4.07
ITCR	0.006	0.023	0.28	TC_t	0.072	0.051	1.42
ITM	0.008	0.023	0.33	DFii_s	0.198	0.013	15.19
PIFL_cd	0	0.075	0.00	DFiii_s	0.183	0.037	4.97
PIFL_cr	0	0.049	0.00	FDL_iii	0.214	0.037	5.78
PIFM_cd	0	0.075	0.00	Mean T/M ratio			7.82
PIFM_cr	0	0.040	0.00				
FCM	0.003	0.74	0.01				
Mean T/M ratio			0.71				

Muscles are organized by group (proximal vs. distal) and mean T/M ratio (TSL/OFL) for each group listed. For full muscle names, see the Supplementary Material.

We addressed three questions: 1) On average, how does activation level interact with MTU compliance, initial passive muscle length, and/or activation-dependent shift in L0 to influence the operating range and force capacity of muscles? 2) Under what combinations do these factors meaningfully influence force generating capacity of an individual muscle? 3) How do these factors sum across several muscles acting across a joint to influence the net force and/or torque generating capacity during a low-activation isometric task like standing?

## Materials and methods

### OpenSim model development

**Fig. 2:**
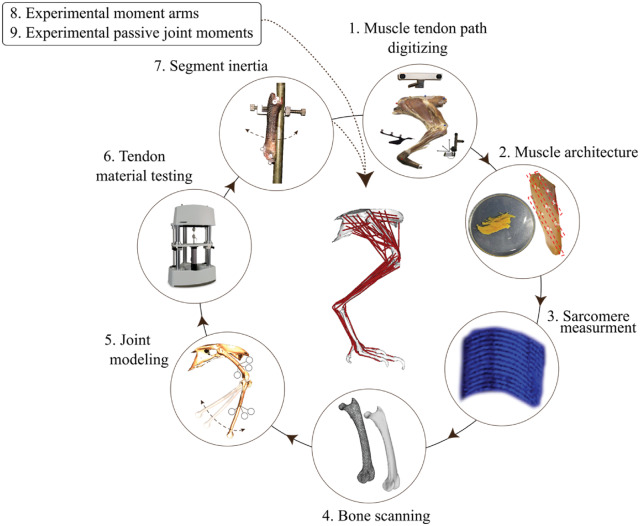
Model development framework and main steps. See the “Materials and methods” section and Supplementary Material for details.

Our approach to explore these questions was to build a realistic detailed musculoskeletal model. Modeling was done first in SIMM software (Delp and Loan 1995) and subsequently converted to OpenSim (Seth et al. 2018). This model incorporated several experimental measurements of muscle and skeletal properties as well as steps to validate the model accuracy (Fig. 2). A detailed description of each modeling step is provided in the Supplementary Material and summarized below.

### Animals

Complete muscle–tendon paths, bone geometry, and muscle architecture measurements were made on one guinea fowl specimen (1.45 kg body mass) to construct a generic musculoskeletal model of the pelvic limb. Four additional animals (1.46 ± 0.1 kg; mean ± SD) were used to compare general muscle and tendon properties (muscle mass, fiber length, pennation angle, tendon length, and mass). Experimental moment arm measurements were performed on two animals (1.55 kg; 1.49 kg) for ankle and tarsometatarsus-phalangeal (TMP) muscles and on four animals (1.59 ± 0.1 kg; mean ± SD; taken from Carr et al. 2011) for the moment arm of the knee extensors (patella) and hip extensor muscle (the ILPO; see muscle abbreviations in Supplementary Table S1). These experimental moment arms were compared with those predicted by the model. *In vivo* passive joint moment experiments were performed on four animals (1.55± 0.2 kg; mean ± SD) and compared with the generic model predictions. Two animals (1.43 kg; 1.49 kg) were used to measure tendon elastic modulus. Animal experiments were performed under protocols approved by the Northeastern University Institutional Animal Care and Use Committee (NU IACUC) and all specimens used only for anatomical measurements were obtained post euthanasia from NU IACUC approved protocols. The model specimen and the additional muscle architecture specimens were transferred to the Stanford University Neuromuscular Biomechanical Laboratory for model development.

Step 1: Muscle–tendon paths: Modeling began by digitizing the 3D muscle–tendon paths of the model animal (1.45 kg). This was achieved by isolating the pelvic limb and systematically dissecting off each muscle–tendon-unit (MTU) from the right limb (kept fresh/frozen). The muscle–tendon path from origin to insertion was traced using an optical tracking system (Polaris, Northern Digital, Waterloo, ON). A total of 39 MTUs were defined for this study (for a complete muscle list of the model see Supplementary Material), with some large muscles being divided into sub-muscles. Care was taken to capture the geometry of MTU paths across articulating surfaces, which were used to define muscle wrapping surfaces in SIMM software.

Step 2: Muscle architecture: Experimental muscle architecture measurements included the muscle mass and free tendon mass and length. The left limb was fixed (10% neutral buffered formalin) in mid-swing posture (Rubenson and Marsh 2009). Muscle fascicle lengths were measured with calipers and took into account curvature of muscle fibers.

Step 3: Sarcomere lengths: Bundles of fascicles were isolated and used for sarcomere length measurements (*L*_s_) based on second harmonic generation using two-photon laser microscopy (Cromie et al. 2013). Optimal muscle fiber lengths (L0) were defined as (Llewellyn et al. 2008) L0=Lf⋅2.36Ls, where 2.36 is the length in microns of the optimal sarcomere length in guinea fowl muscle (Carr et al. 2011). The cross-sectional area (CSA) of the muscle was calculated as from the L0, muscle mass (*m*_mus_) and density ($\rho_{\mathrm{mus}}$, 1060 kg/m^3^) as: $\mathrm{CSA}=\;\frac{m_{\mathrm{mus}}}{\rho_{\mathrm{mus}}\cdot L_{0}}$. CSA rather than physiological CSA was calculated since pennation angle is a separate parameter in muscle models in OpenSim. Maximal isometric force (*F*_100max_) for each muscle unit was calculated using a specific tension of 3e5 N/m^2^.

Step 4: Bone geometry: Skeletal elements were 3D-scanned after cleaning to generate bone mesh (.ply) files. Pelvis, femur, and tarsometatarsus were scanned individually, and the phalangeal segments were scanned together and separated in software by estimating the location of center of rotation between adjacent segments.

Step 5: Joint modeling: Anatomical and functionally relevant bone coordinate systems (B-ACS) were defined, incorporating mathematically derived joint centers and axes. For the knee, ankle, and tarsometatarsus-phalangeal (TMP) joint, a helical axis and functional joint center were computed from 3D motion capture data of adjacent segment motion (Rubenson et al. 2007). Ankle joint translation and patella-complex motion were defined as a function of ankle and knee joint angle, respectively (measured from 3D motion capture). The motion of the patella in this study was performed on an anatomical specimen but appears qualitatively similar to the motion of the patella recorded *in vivo* in locomoting guinea fowl (Allen et al. 2017). The approach used in the present study to compute extensor moment arms of muscles attached to the patella (virtual work approach) differed in approach to that of Allen et al. (2017; geometric approach). The hip joint center was defined by directly digitizing its location with the 3D pointer. The bone model files and muscle–tendon paths were transformed into the relevant B-ACSs that were used to define the final model’s joint coordinate systems (JCSs; see Supplementary Material for a full definition of B-ACSs and JCSs).

Step 6: Tendon properties: We measured tendon material properties from two separate animals (1.43 kg; 1.49 kg) of the free common tendon of the lateral, medial, and intermedius gastrocnemius muscles (Achilles), and the free tendon from the tibialis cranialis, digital flexor IV, and extensor digitorum longus (Bose EnduraTEC, ELectroForce 3200, Framingham, MA). The tendons were programmed to undergo a 5 Hz sinusoidal cycle that approximated the loading duration of the stance phase and swing phase of moderate running speeds (∼2.0 m/s), where stance and swing times are similar (∼200 ms; (Gatesy 1999; Ellerby and Marsh 2006; Rubenson and Marsh 2009). The clamp displacement was programmed to produce force approximating *F*_100max_. These data were used to generate muscle-specific tendon load-elongation curves for the tested muscles and to generate a generic (average) load-elongation curve for the remaining tendons. The tendon slack lengths were solved for by adjusting the slack length of each tendon until the passive simulated muscle fiber length of the model matched the experimental fiber length in joint postures matching the experimental specimen. Using the equality between a) the output MTU length from the model at the fixed specimen’s joint angles (based on geometry; MTU_osim_) and b) the experimental MTU length computed from individual muscle and tendon lengths (MTU_exp_) at these same joint postures, the tendon slack length can be solved for using a root solver (MATLAB, fsolve) also of the function:

$${\mathrm{MTU}}_{\mathrm{osim}}-{\mathrm{MTU}}_{\exp}=0.$$

In this function, MTU_exp_ is set as:

$${\mathrm{MTU}}_{\exp}=\;\left({\mathrm{Lm}}_{\exp}\cdot \cos\theta \right)+\mathrm{Lt},$$

where ${\mathrm{Lm}}_{\exp}$ and cos⁡θ are the experimentally measured fiber length and pennation angle from the fixed specimen, respectively, and Lt is the length of the tendon. Lt is in turn defined by the sum of the tendon slack length (${\mathrm{Lt}}_{\mathrm{slack}}$) and any stretch present in the tendon (${\mathrm{Lt}}_{\mathrm{stretch}})$:

$${\mathrm{Lt}={\mathrm{Lt}}_{\mathrm{slack}}+\;\mathrm{Lt}}_{\mathrm{stretch}},$$

$${\mathrm{Lt}}_{\mathrm{stretch}}\;\text{is}\;\text{computed}\;\text{as}:$$

$${\mathrm{Lt}}_{\mathrm{stretch}}={\mathrm{Lt}}_{\mathrm{slack}}\cdot \;f_{\theta \epsilon }\left(F_{p},\;F_{100m\mathrm{ax}}\right),$$

where $f_{\theta \epsilon }$ is the normalized tendon stress–strain spline function (see Supplementary Material) that predicts tendon strain using passive tendon force and the maximal isometric muscle force (*F*_100max_) as input.

The passive tendon force $F_{p}$ is computes as:

$$F_{p}=\;f_{\mathrm{fl}}\left(Lm_{\exp},\;F_{100m\mathrm{ax}}\right)\cdot \cos\theta ,$$

where $f_{\mathrm{fl}}$ is the normalized passive muscle F–L spline function that predicts passive muscle force using muscle fiber length and *F*_100max_. Combining equations, a root function for solving ${\mathrm{Lt}}_{\mathrm{slack}}$ can be defined requiring only the output MTU length from OpenSim, the experimental muscle fiber length and pennation angle at the corresponding joint posture, and the predicted maximal isometric force of the muscle:

$${\mathrm{MTU}}_{\mathrm{osim}}-\left[\left({\mathrm{Lm}}_{\exp}\cdot \cos\theta \right)\right]-\left[{\mathrm{Lt}}_{\mathrm{slack}}\right]-\left[{\mathrm{Lt}}_{\mathrm{slack}}\cdot \;f_{\theta \epsilon }\left(F_{p},\;F_{100m\mathrm{ax}}\right)\right]\;=0.$$

Step 7: Segment inertial properties: Segment inertial properties for the model were obtained from previously collected data from six animals (see Rubenson and Marsh 2009). In short, limb segments were disarticulated, weighed, and frozen. The center of mass of each segment was determined by suspending the segment twice from different attachment points. The plumb line of the string for each suspension was transformed into the bone coordinate system. The center of mass was the point where the two plumb lines intersect (Alexander 1968). The moment of inertia of each segment was measured using a pendulum method. A small hole was drilled through the proximal or distal end of the frozen segment, allowing the segment to rotate around a steel rod (3-mm diameter). The segment was perturbed, and its period of oscillation was determined by videoing the segment. The inertia of the segment about the axis of rotation was calculated using the parallel axis theorem and the distance from the point of rotation to the segment center of mass (Rubenson and Marsh 2009).

Step 8: Muscle moment arms: Muscle moment arms were computed from tendon travel experiments measured post mortem in separate specimens following an approach described previously (Carr et al. 2011). In short, to quantify the moment arm for any given muscle across a joint, we combined simultaneous recordings of tendon length and joint angle as the ankle, knee, or hip was rotated through its range of motion. For all muscles, the length transducer lever was counterweighted to ensure that there was no slack in the suture and that any small strain in tendon (for the ankle muscles) or suture was kept constant (see Supplementary Material for additional details).

**Fig. 3:**
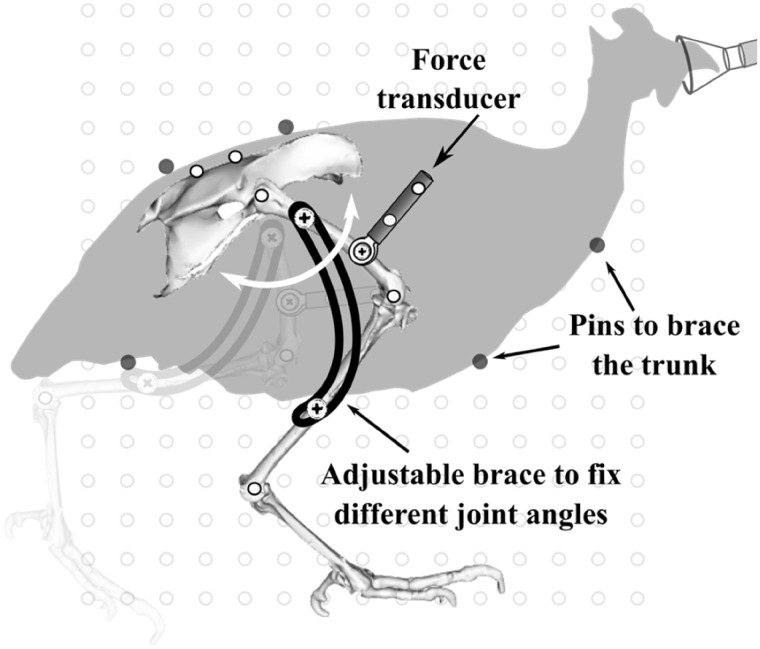
Experimental set-up for passive net joint moment measurements. Example shown for passive hip joint measurement. Also performed for ankle and TMP joints.

Step 9: Passive net joint moments: Passive net joint moments were measured for the hip joint (proximal muscles) and ankle joint (distal muscles) from deeply anesthetized and nerve-blocked animals, separate to that of the model specimen (1.55 ± 0.2 kg; mean ± SD). We used a custom limb immobilization rig with the animal positioned on its side that allowed us to freely rotate the joint of interest while immobilizing the adjacent joints at set angles (Fig. 3). We attached a single-axis compression/tension quartz force transducer (Kistler model 9203) to the bone distal to the joint of interest using a stainless-steel mounting screw. The joint of interest was held horizontal and rotated through its flexion/extension range of motion by pushing/pulling the transducer. Force was recorded continuously at 1000 Hz and the origin and orientation of transducer was identified by video recording of reflective markers on the sensor (Fig. 3). The skeletal planar kinematics were recorded from reflective markers placed on joint centers and bone landmarks following the procedures published earlier (Rubenson and Marsh 2009). The force transducer and joint kinematics were synchronized using a TTL pulse that was recorded on a separate A-to-D channel and that simultaneously turned on an LED in the video field of view. Additional details of these experiments and results are presented in the Supplementary Material.

Finally, to assess the accuracy of our model we compared simulated data with experimental values for both muscle moment arms and passive net joint moments. Results of these comparisons can be found in the Supplementary Material.

### Computation of fiber lengths during isometric (fixed-posture) contractions

In this musculoskeletal model, we performed forward simulations of fixed-end contractions across a range of prescribed activation levels and limb configurations (Fig. 1B) intended to elicit the maximum *in vivo* range of muscle lengths. MTU lengths were roughly prescribed by setting a posture that set an initial passive length (short, medium, and long) and then imposing smaller deviations in MTU lengths around that initial position through variation of either the ankle (distal muscles) or hip angle (proximal muscles) through 95% of the range for that joint during locomotion and jumping reported in the literature across various experiments (Henry et al. 2005; Ellerby and Marsh 2006, 2010; Rubenson and Marsh 2009). Each posture generated a range of passive MTU lengths across the limb determined by fiber length, moment arm, and tendon slack length of each muscle. At each of these postures we implemented three different muscles models (1: No Compliance, No activation-dependent shift in L0 [NCNS], 2: Compliance with No activation-dependent shift in L0 [CNS], and 3: Compliance with a 15% activation-dependent lowercase shift in L0 [CS] and simulated fixed-end contractions at five activation levels [0%, 25%, 50%, 75%, 100%]). Simulations for muscle models without activation-dependent shift were performed on the unaltered model with and without tendon compliance. Including activation-dependent shifts of optimal fiber length required altering the F–L curve for activation levels below 100%. For each activation level, the F–L curve for each muscle was replaced in the OpenSim model with a shifted curve (see Supplementary Material for details). A 15% activation-dependent shift in L0 (at zero activation) was used because it represents a typical value reported in the literature for both voluntary muscle activation and electrically stimulated isolated muscle under realistic stimulation frequencies (Rack and Westbury 1969; Roszek et al. 1994; de Brito Fontana and Herzog 2016), and also because it is a value previously adopted in modeling of muscle force (Lloyd and Besier 2003; Buchanan et al. 2004). For each simulation, we extracted the normalized active individual fiber force and normalized fiber length for each muscle. Passive muscle lengths were defined as the muscle length at 0% activation, resulting in 46,800 MTU lengths (Fig. 1B). These results were analyzed in three ways.

#### 1. On average, how does activation level interact with MTU compliance, initial passive muscle length, and/or activation-dependent shift in L0 to influence the operating range and force capacity of muscles?

To discern whether there are any useful broad patterns of interaction between MTU compliance (i.e., the ratio of tendon slack length to optimal fiber length, Table 1), initial passive muscle length, and activation-dependent shifts in L0, we binned data into two levels of compliance (Low < 2 < High), and passive muscle length (ascending [A], plateau [P], or descending [D] limb of the F–L curve) and evaluated how muscle length changed with activation level between muscle models. For each group (Table 1), we calculate average normalized muscle length [*L*/(L0)] and force (*F*/*F*_100max_) across all muscles in each group (Table 2) at each level of activation (0%, 25%, 50%, 75%, 100%). Additionally, at each activation level we calculated how muscle length changed with activation (Δ*L*) from its passive starting length. For instance, Δ*L* at 50% activation would be given by

$$\Delta L\;=\;L_{A50}-L_{A0},$$

where the muscle length at 50% and 0% activation (normalized to L0 at 100% activation) for this passive length is given by *L*_A50_ and *L*_A0_, respectively. Main and interaction effects were evaluated with a nested linear mixed effects model (Bates et al. 2015) with Δ*L* as the dependent variable, muscle as a random factor and activation level (0, 25, 50, 75, 100), activation-dependent shift in L0 (0, 15%), and passive length of the muscle (A, P, D) as independent factors. Given that activation-dependent shifts in the optimal fiber length did not show a main or any interaction effects, the statistical models were run both with and without this factor. Throughout this paper, muscle lengths are reported as normalized by the optimum fiber length at 100% activation, L0, and forces by maximum force at 100% activation, *F*_100max_.

**Table 2:** Triple nested experimental design

	MTU compliance (T/M ratio)	Low			High		
	Passive length (*Ls*)	Ascending	Plateau	Descending	Ascending	Plateau	Descending
Activation-dependent shift in L0 (*S*_LO_)	0%	1, 25, 50, 75, 100	1, 25, 50, 75, 100	1, 25, 50, 75, 100	1, 25, 50, 75, 100	1, 25, 50, 75, 100	1, 25, 50, 75, 100
	15%	1, 25, 50, 75, 100	1, 25, 50, 75, 100	1, 25, 50, 75, 100	1, 25, 50, 75, 100	1, 25, 50, 75, 100	1, 25, 50, 75, 100

#### 2. Under what combinations of these factors is the force generating capacity of an individual muscle meaningfully altered? Specifically, we asked how compliance and activation-dependent shift in L0 alter muscle operating length and force capacity at high (100%) and low (25%) activation and which combinations of these factors alter the relative force capacity substantially.

Our second analysis aimed to quantify our results at a finer level of detail. While in the first analysis we binned our data into two compliance levels and three different passive length ranges, here we present the length and force data for each muscle at all simulated passive lengths (9216 iterations). Again, we isolated the influence of compliance and activation-dependent shifts in L0 by comparing resultant muscle lengths and forces between the three muscle–tendon models (NCNS, CNS, and CS). Differences between models with and without compliance isolate the influence of compliance alone. Likewise, differences between models with compliance but with and without activation-dependent shifts in L0 allow us to isolate the contribution of activation-dependent shift to changes in operating length and force capacity. Specifically, the contribution of activation-dependent shift in L0 to muscle operating length (Δ*L*) for a given muscle, passive length, and activation level was found by subtracting the muscle length of the CNS condition, *L*_CNS_, from that of the CS condition, *L*_CS_.

$${\Delta L}_{\mathrm{ADshift}}=\;L_{\mathrm{CS}}-L_{\mathrm{CNS}}.$$

The force values for each muscle with no compliance or shift (i.e., non-compliant tendon, NCNS) were found by assuming no change in muscle length across activation levels and scaling the force with activation. For example, for a muscle with a passive length of 0.809 at 100% activation, *F*_100max_, is 0.726. At 50% activation, the calculated force with no compliance would be 0.363 or

$$F_{\text{5}0}\;=\;0.\text{5}\;*\;F_{\text{1}00\text{max}}$$

**Fig. 4:**
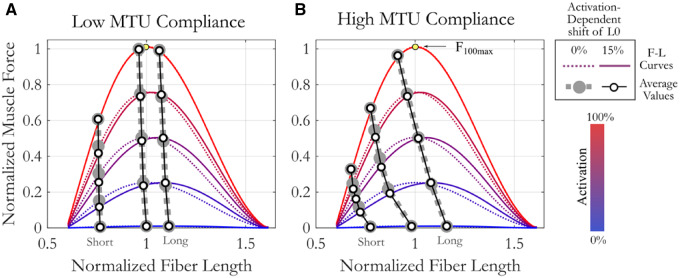
The influence of compliance level (low [**A**], high [**B**]) on average muscle operating length and force capacity across activation levels. On average, muscle length decreases with increasing activation and is significantly influenced by interactions between starting length, compliance, and activation level. Muscles with low MTU compliance (A) change length with activation less than muscles with high compliance (B). While activation-dependent shifts in L0 change operating length (difference between hollow and filled circles), they are not significant. F–L curves at five different activation levels are distinguished by color (blue: low activation, red: high activation). Line type designates presence (solid) or absence (dashed) of activation-dependent shift in L0. Circles represent the mean value at the five different activation levels for muscle models with (hollow) or without (filled) activation-dependent shift in L0. L0_100_ is always a normalized fiber length of 1. Yellow circle marks the maximum force at 100% activation, *F*_100max_.

Since the significance of a change in muscle length or force will vary by context, we chose to present these data in two ways, as absolute changes normalized by maximum force capacity at 100% activation (*F*_100max_) and as relative changes between models. Absolute changes were plotted for all data points as a function of MTU compliance and passive length normalized to muscle length at 100% activation, L0_100_ (Fig. 4). Absolute changes relative to maximal force allowed us to quantify the contribution of each factor as well as painting a more detailed picture of the interaction between these factors and the conditions in which each is most influential. Yet, this approach can obscure the significance of changes at low activations. For instance, at 25% activation, a change in force capacity of 5% of maximum force between models may represent a relative change of anywhere from 20% to 80% of force capacity. To capture these relative effects, we again binned our data, but this time into much smaller bin sizes (steps of 5% of L0, T/M ratio bins: 0.2 for low compliant muscles and 1 for high) and compared across models as described above (NCNS, CNS, and CS). For each bin we calculated the number of samples in each bin and the percent of samples with a >10% difference in force capacity between models. The 10% difference is an arbitrary cut-off below which we deemed the activation-compliance effect to be of less importance in dictating muscle function. The percent difference between muscle–tendon models was taken from the difference between the forces predicted by each model divided by the force predicted by the less complex model. For example, the percent difference between models with and without compliance (NCNS and CNS) was given by

$$\%\Delta F\;=100*\;\frac{{F_{\mathrm{CNS}}-F}_{\mathrm{NCNS}}}{F_{\mathrm{NCNS}}},$$

where *F*_CNS_ and *F*_NCNS_ are the forces predicted by the CNS model and NCNS models, respectively. With this analysis we aimed to identify a threshold of MTU compliance or range of operating lengths where these factors can be safely ignored and the conditions in which simple models that exclude these factors lead to errors.

#### 3. What are the functional consequences of these factors? Specifically, how do these activation-dependent factors sum over many muscles acting across a joint to influence the posture for maximal force and moment capacity at low activation (25%) and how does this influence compare between a distal and proximal joint?

**Fig. 5:**
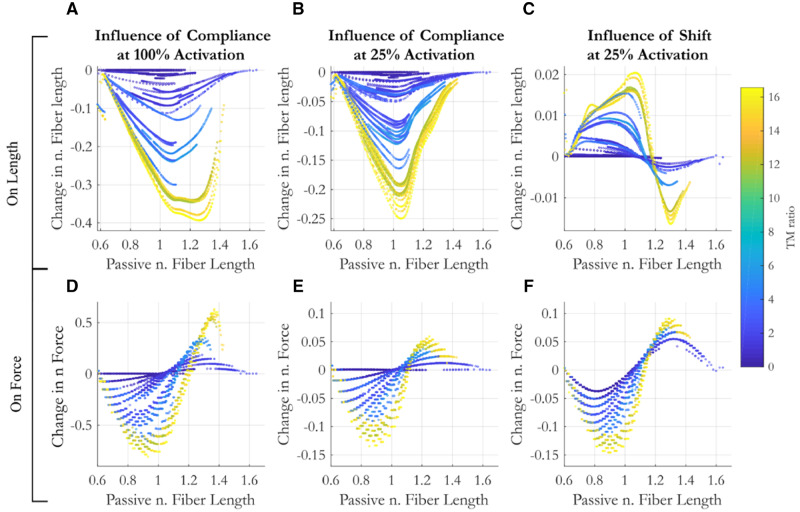
The influence of compliance (**A**, **B**, **D**, **E**) and an activation-dependent shift (**C**, **F**) in the optimal fiber length on muscle length (A–C) and force (D–E) as a function of the passive (pre-activation) fiber length and MTU compliance (designated by color).

To explore these questions, we compared force and torque generated by the sum of muscles acting across the ankle and the hip. We chose to evaluate the muscles acting across the hip and ankle since on average they are composed of MTUs with very different levels of compliance (T/M Ratio Hip: 0.71, Ankle: 7.82, Table 2) and did not overlap. We chose a consistent 25% activation to match the lowest level of activation used to evaluate our second question above and made it uniform to simplify the analysis. We simulated 25% activation of muscles acting across these joints for each model (NCNS, CNS, and CS) through a sweep of hip and ankle postures with knee and TMP angle held constant at standing angles (Ankle and hip angle ranges as described in Fig. 1. Knee_flex_: −110°, TMP_flex_: −55° [Henry et al. 2005]). For each joint angle we extracted the total force and torque acting across each joint as well as the mean moment arm weighted by maximal muscle force, *F*_100max_ (Fig. 5). Maximum total muscle force and torque were found for each joint for each model as well as the corresponding joint angle (Table 4). For reference, we then calculated the percent difference between these joint angle-dependent values and the force or torque in the standing posture. The goal of this analysis was to provide an estimate of the combined influence of the activation-compliance dependent effects across many muscles at low activation.

**Table 4:** A comparison of functional influence of three different muscle–tendon models: No compliance No Shift (NCNS), Compliance but No Shift (CNS), and Compliance and Shift (CS)

	Hip T/M ratio: 0.71							Ankle T/M ratio 8.46						
	Max values				Standing 50°			Max values				Standing 98°		
	Force		Moment		*n*FL	*F* (N)	*M* (Nm)	Force		Moment		*n*FL	*F* (N)	*M* (Nm)
	Mag	°	Mag	°				Mag	°	Mag	°			
NCNS	65.8	50	0.67	24	0.97	65.8	0.52	75.2	64	0.65	68	1.21	60.2	0.53
CNS	62.1	56	0.62	20	0.95	62.0	0.44	77.4	90	0.67	88	1.06	76.3	0.65
CS	61.4	72	0.58	28	0.95	59.8	0.48	78.2	100	0.66	96	1.05	78.1	0.60

For each model, the joint angle (°) and magnitude (Mag) at maximum force and moment across all joint angles at the hip (low compliance) and the ankle (high compliance) are provided. This can be compared with total force (*F*) and moment (*M*) at each joint in a standing posture. The average normalized fiber length (*n*FL) of muscles acting at each joint in each condition highlights the influence of the different muscle models on operating length. Compliance and shifts in L0 decrease the force and torque capacity at the hip while increasing them at the ankle.

## Results

### 1. On average, how does activation level interact with MTU compliance, passive muscle length, and/or activation-dependent shifts in optimal fiber length to influence the operating range and force capacity of muscles?

We found passive muscle length, activation, and MTU compliance influenced how a muscle changes length with increasing activation, while activation-dependent shifts in optimal fiber lengths did not (Fig. 4 and Table 3). There were neither significant main nor interaction effects with activation-dependent shifts in L0. When models were re-run without including activation-dependent shifts in L0, we found third-order interaction terms between passive muscle length, activation level, and MTU compliance (Table 3). These interaction terms imply that how muscle length changes with activation level differs between muscle of low and high compliance and that this relationship changes with the starting passive length of the muscle.

**Table 3:** Model parameters and results for linear mixed effects models with change in muscle length as dependent factor

	Shift * T/M ratio * Limb * Act					T/M ratio * Limb * Act			
Model	*DF*	Sum sq.	Mean sq.	*F*-value	*P*-value	Sum sq.	Mean sq.	*F*-value	*P*-value
Shift (1)	1	0.02	0.02	17.4	0.15				
T/M ratio (2)	1	0.94	0.094	83.1	0.07	0.097	0.097	83.08	0.07
**Limb (3)**	**2**	**13.07**	**6.53**	**5768.2**	**1.7e−4**	**6.8**	**3.3**	**2808.9**	**3.6e−4**
**Act (4)**	**4**	**83.87**	**20.96**	**18502.8**	**1.75e−8**	**41.9**	**10.5**	**8964.4**	**7.4e−8**
1×2	1	0.017	0.167	14.70	0.01				
1×3	2	0.016	0.008	7.03	0.01				
**2**×**3**	**2**	**5.95**	**2.97**	**2625.2**	**3.08e-4**	**3.04**	**1.5**	**1300**	**7.7e-4**
1×4	4	0.018	0.005	4.07	0.1				
**2**×**4**	4	**39.83**	**9.96**	**8787.3**	**7.8e−8**	**19.98**	**4.99**	**4265.6**	**3.3e−7**
**3**×**4**	8	**14.49**	**1.81**	**1598.6**	**1.6e−10**	**7.5**	**0.94**	**804.5**	**2.6e−9**
1×2×3	2	0.005	0.002	2.27	0.2				
1×2×4	4	0.013	0.003	2.95	0.16				
1×3×4	8	0.022	0.003	2.37	0.16				
**2**×**3**×**4**	**8**	**5.78**	**0.722**	**637.4**	**6.4e−9**	**3.02**	**0.38**	**322.1**	**9.5e−8**
1×2×3×4	8	0.008	0.001	0.89	0.3				

Activation-dependent shift in L0 (Shift) did not have significant main or interaction effect. Activation level, MTU compliance (TFR), and passive region of the F–L curve all interact to influence operating muscle length. Bolded values highlight models with parameters that significantly influenced muscle length.

This complex interaction can be seen in Fig. 3. While both high and low compliance muscles become shorter with increasing activation, high compliance muscles show a larger effect. The extent of the effect is also dependent on passive muscle length. Muscles with passive lengths on the descending limb of the F–L curve showed larger length changes with increasing activation for all levels of compliance (Fig. 4). This effect is explained on the basis that force capacity on the descending limb, and thus the compliance effect, increases as the muscle shortens toward L0.

### 2. Under what combinations of these factors is the force generating capacity of an individual muscle meaningfully altered? How does compliance and activation-dependent shifts in optimal fiber length alter muscle operating length and force capacity at high (100%) and low (25%) activation?

The influence of compliance: More compliant muscles show greater changes in length across activation levels. At 100% activation (Fig. 5A), the change in length can be as high as 40% fiber strain while at 25% activation that decreases to 25% fiber strain (Fig. 5B). At high activation, the starting passive length which results in the greatest length changes increases with MTU compliance (Fig. 5A, shifting from ∼1.0 to 1.25 L0) while at low activations the passive length that produces the greatest length changes is less influenced by compliance (Fig. 5B, shifting only from ∼1.0 to 1.05 *L*0). The consequence of these length changes on the force capacity depends on the passive muscle length, generally hampering force generation at short passive lengths and amplifying force capacity at long passive lengths (Fig. 5D). Although it is interesting to note that change in force capacity does not linearly scale with passive muscle length. At the tails of the F–L curve (short and long extremes), the force effects are smaller due to the decreased force capacity. Thus, the passive length that results in the greatest change in muscle length does not occur at the extremes nor does it align with the length that results in the greatest change in force. The transition between decreasing and increasing force capacity as a result of activation compliance effects occurs at longer passive muscle lengths for more compliant muscles. At 100% activation, the transition shifts from a normalized length of 1 at low compliance to 1.2 for high compliant muscles. The results at 25% activation, again, show similar patterns, but with less dramatic variations (Fig. 5E).

The influence of activation-dependent shifts in optimal fiber length: In comparison to the influence of compliance, activation-dependent shifts in optimal fiber length result in much smaller changes in normalized muscle length. Muscle lengths change by at most 2% of *L*0_100_ (Fig. 5C). Unlike the influence of compliance, though, shifts in optimal fiber length result in longer as well as shorter muscles depending on passive muscle lengths. While intuition may suggest that activation-dependent shifts alone would not alter a muscle’s length, the variations are likely the result of muscles finding an equilibrium with the compliant tendon. For a muscle on the ascending limb of the F–L curve, for instance, a shift in the F–L curve to the right decreases its force capacity, resulting in less stretch of the tendon. Thus, for any fixed muscle–tendon length, the equilibrium length of the muscle on the ascending limb is longer in the presence of activation-dependent shifts. The opposite effect can be seen on the descending limb (Fig. 5C), and again, these effects are amplified for more compliant MTUs.

In contrast to the influence of compliance, which has little effect on the force generating capacity of low compliance muscles, activation-dependent shift in *L*0 influences the force capacity of muscles of all compliance levels, though the greatest effects are still seen in the most compliant MTUs. Activation-dependent shifts in *L*0 can result in both decreased and increased force capacity, depending on passive muscle length and compliance. In general, muscles with passive lengths less than ∼1.1 *L*0 decrease force capacity in the presence of activation-dependent shifts while muscles above a normalized length of 1.1 *L*0 increase force capacity. The magnitude of the change in force capacity with shift matches or exceeds the influence of compliance at 25% activation.

In summary, at high activation, compliance results in muscle strain up to 40%. These changes result in increases of force capacity for muscles on the descending limb of the F–L curve (up to 60% of maximum force, *F*_100max_) and decreases on the ascending limb (of up to 80% of maximum muscle force capacity). Muscle force is most sensitive to compliance effects on the shallow ascending limb (*Ls* ∼ 0.7–0.9) or the middle descending limb (*Ls* ∼ 1.2–1.3) of the F–L curve. At low activations, though activation-dependent shifts in L0 result in only small changes in muscle length (at most 1–2% strain), they have a larger effect on force capacity than compliance (Fig. 5F vs. Fig. 5E) since the peak of the F–L curve is changing relative to the muscle length. While the influence of activation-dependent shifts in *L*0 on force capacity decreases with compliance, for muscles with no tendon, they can be as high as 5% of maximum force capacity.

#### Which combinations of these factors alter the relative force capacity by more than 10%?

**Fig. 6:**
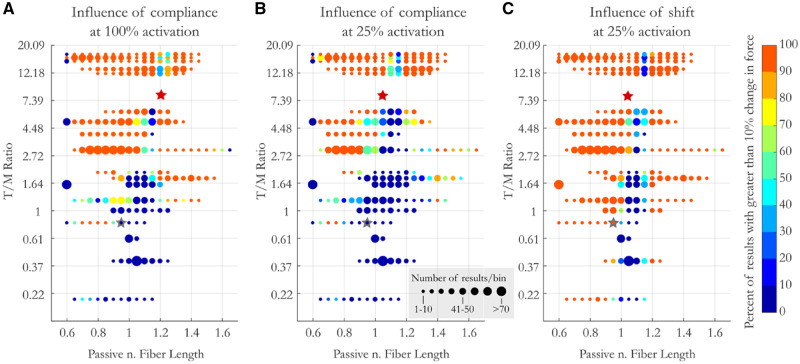
The influence of compliance (at 100% [**A**] and 25% activation [**B**]) and activation-dependent shifts in L0 on force production (at 25% activation [**C**]) as a function of MTU compliance and passive (pre-activation) muscle length. Color designates the percent of results that saw a greater than 10% change in force production between models. Circle size represents the number of samples per bin. *Y*-axis is non-linear to ease visualization of results at low MTU compliance. Stars represent average compliance and operating length of the muscles acting at the hip (gray) and ankle (red) in CNS (B) and CS (C) conditions.

Influence of compliance: The range of passive lengths that result in a change of force capacity increases with compliance and activation, as expected. For the most compliant MTUs, 88% of starting passive muscle lengths result in a change in force capacity that is >10% (Fig. 6A). While our data does not allow us to fully quantify the space, it does allow us to isolate regions of high and low sensitivity to compliance. For instance, at both 100% and 25% activation, *all* MTUs with a T/M ratio as low at 0.8 show a meaningful (>10%) influence of compliance on force capacity when the muscle’s initial passive length is between 0.7 and 0.75 *L*0 (Fig. 6A, B). Thus, even low compliance MTUs have a region of operating lengths where compliance meaningfully influences force capacity. There also exists a trough of influence where compliance and activation-dependent shifts of *L*0 have little influence on force capacity. The location of this trough varies with MTU compliance, though, shifting slightly to longer muscle lengths with increasing compliance. For example, MTUs with a T/M ratio of 1.2 show no change in force capacity with compliance when operating at 1.05 *L*0, while the passive length that has the smallest influences on the force capacity of the most compliant muscles is closer to 1.15 at 25% activation and 1.25 at 100% (Fig. 6C).

Influence of activation-dependent shifts in L0: Unlike compliance, activation-dependent shifts alter the force capacity of muscles of all levels of compliance. While compliance had very little influence on the force capacity for muscles with very little tendon, activation-dependent shifts in *L*0 resulted in changes in force capacity of over 10% for muscles with no tendon (T/M ratio = 0) across half of the operating range, primarily at lengths on the ascending limb. Again, the influence increased with increasing compliance, such that for the most compliant muscles activation-dependent shifts in *L*0 resulted in changes in force capacity for all muscles across 95% of the possible initial passive lengths of the muscle. The trough of influence for activation-dependent shifts in *L*0 followed similar patterns to that for compliance, with minimal effect on force for stiff MTUs at lengths of 1.05 and for compliant muscles at 1.15.

To summarize, at high activation compliance considerably changes the force capacity for muscles with a T/M ratio over 2 at nearly all passive lengths (95%). While change in force capacity increases with compliance, all muscles with low compliance (i.e., T/M ratio as low as 0.7) are also affected when passive muscle lengths are in the middle of the ascending limb (Lp: 0.7–0.85). At 25% activation, the results are similar but with decreased magnitude. Activation-dependent shifts considerably influence the force capacity of all muscles on the ascending limb of the F–L curve. For all of these effects, there is a trough of influence near or slightly above *L*0 where activation does not alter force capacity appreciably.

### 3. How do these compliance and activation-dependent factors influence the posture which maximize the force generating or moment capacity?

**Fig. 7:**
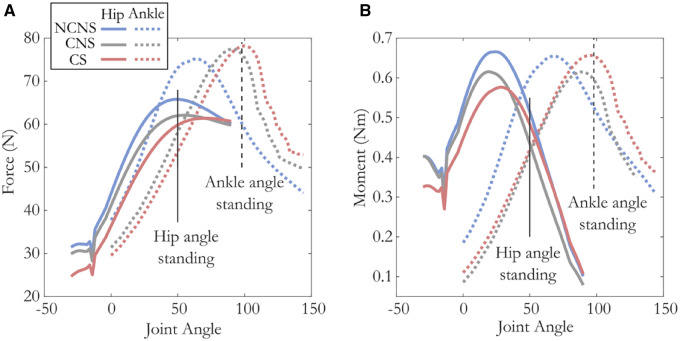
Joint angle vs. total force (**A**) and moment (**B**) acting across the hip (solid lines) and ankle (dashed lines) under three different muscle–tendon models. Average weighted moment arms for the hip (solid) and ankle (dashed) depicted in orange. While at the hip, the posture that produces maximum force and moment does not vary much between models (<9°), the maximum force and moment capacity decreases with both compliance and activation-dependent shift in L0 (∼13%). In contrast, at the ankle the magnitude of maximum force and torque varies little between models (<3%), but the posture at which this maximum occurs varies by almost 40°. Vertical lines represent the hip (solid) and ankle (dashed) postures during standing.

Influence of compliance: As expected, compliance has less of an effect on the force capacity for muscles acting at the hip than for more distal muscles acting at the ankle. At the hip, the postures that maximize force and moment capacity change little with compliance (4° and 6°, respectively). In contrast, at the ankle the postures that maximize force and moment capacity change by 26° and 20°, respectively (Fig. 7A, B and Table 4). Likewise, we see very little difference in force capacity at the hip between models that do and do not include compliance in a standing posture (<−6%, blue vs. gray solid lines at 50°; Fig. 7A). When muscle moment arms are taken into account we observe a reduction in moment capacity (−15%) compared with maximum values at a more extended posture. At the ankle, muscles operating on the descending limb of the F–L curve in a standing posture benefit from the change in length with compliance such that they approach *L*0 and increase their force capacity by 27% and joint moment by +23% (CNS model; blue vs. gray dashed lines at 98°; Fig. 7A).

Influence of activation-dependent shifts in L0: The influence of activation-dependent shifts in L0 shows a different pattern than that of compliance. Both the hip and ankle are equally influenced by activation-dependent shifts in L0. At the hip, the postures that maximize force capacity and moment change by 16° and 8° when activation-dependent shifts in L0 are included in the model, while the ankle postures that maximize force capacity and moment change by 8° and 10° (Fig. 7A, B, respectively, Table 4).

In a standing posture, muscles acting at the hip, since they operate on the ascending limb, are shifted further away from optimal length when an activation-dependent shift in L0 is added to the model. Since they are shifted further from the optimal length, muscles acting at the hip show a decrease in force and moment capacity (Δforce: −4%, Δmoment: −9%, Fig. 7A solid red line). The ankle, though, operates on the plateau of the F–L curve (∼1.06) and its force capacity increases slightly (Δforce: 1.5%, Δmoment: +9%) when an activation-dependent shift in L0 is added to the model (Fig. 6A, dashed red line).

In summary, at the hip, compliance decreases force capacity slightly more than activation-dependent shifts in L0 (−5% and −4%, respectively) in a standing posture, with both effects being small. At the ankle, compliance increases the force generated at the ankle substantially (+26%), while activation-dependent shifts have a much smaller effect (+2%). Similar effects are seen for moment generating capacity.

## Discussion

The F–L relationship is one of the key components affecting a muscle’s capacity to produce force. And while there are multiple factors that are known to influence a muscle’s F–L operating range, their contribution and possible interactions have not been well quantified. Here we tested the effect of, and interaction between, muscle–tendon compliance, activation and activation-dependent shifts in L0 on muscle operating length, and force during simulated fixed-end contractions in architecturally diverse muscles across a multitude of conditions.

### General effect of compliance and activation-dependent shifts in L0 on muscle length and force capacity

One of the overarching goals for this study was to identify general principles (if any) that describe how compliance and activation-dependent shifts in L0 influence muscle operating lengths. While the relationships were quite complex (see the following section), we reveal several notable patterns. First, we found that in this species, on average, most muscles operate on the plateau or ascending limb of the F–L curve irrespective of the level of tendon compliance or activation-dependent shifts in L0. These averages incorporate data across the total possible *in vivo* length range of the muscles, including both the full range of passive lengths and activation levels. Because our analysis spans muscle force levels from near zero to maximum values, we do not expect that F–L ranges would deviate from this in dynamic contractions, with the possible exception of eccentric contractions under maximal activations where force can exceed *F*_100max_ (Herzog et al. 2016). This comprehensive simulation of muscle operating lengths corroborates much of the literature showing that muscles typically do not operate on the descending limb of the F–L curve (Burkholder et al. 2001; Rubenson et al. 2012; Holt and Azizi 2016). This provides further support for the idea that an animal’s neuro-musculoskeletal structure is most often organized to operate on the ascending limb or plateau regions where muscles are inherently more stable and less susceptible to stretch-induced injury (Herzog et al. 1991, 1992; Lutz and Rome 1996; Lindstedt et al. 2001; Morgan et al. 2002).

Our results also show that the most common effect of compliance is a leftward shift on the F–L curve leading to shorter muscle lengths and a decrease in force capacity. Since muscles, on average, operate on the ascending limb, increasing activation shortens muscles further below L0 as the tendon stretches with greater force generation. The exception is for muscles that start at passive lengths longer than L0. Under this condition, the result is an increase in force capacity as the compliant muscle’s fibers shorten toward L0, though some muscle lengths can shorten beyond L0, first increasing and then decreasing force capacity. It is also notable that the average effect of activation-dependent shifts in L0 generally mirrors those described above for compliance, although with much smaller effects. Thus, on average, the effects of compliance dominate activation-dependent changes in operating lengths.

Finally, by comparing results that average across muscles acting at the hip (low compliance) with those acting at the ankle (high compliance), we show that more compliant tendons will generally amplify the effects described above. Given the proximal–distal gradient of compliance, this analysis suggests that proximal muscles will show little activation-compliance effect whereas distal muscles will be more significantly influenced. In summary, if specific knowledge of a muscle’s fiber, tendon and joint characteristics are lacking, the average data provide a cursory prediction of the most likely influence of compliance and activation-dependent shifts in L0 on muscle F–L operating ranges. As muscles increase activation, they will shorten further away from L0 and generate less force than would be predicted by a linear force–activation relationship. This effect increases with compliance and muscle passive length (up to L0). Activation-dependent shifts in L0 amplify this effect, but only minimally. However, these generalizations should be used with caution because, as we will detail in the following sections, they are error prone and can obscure the highly variable effect of compliance and/or activation-dependent shifts in L0 on functionally-relevant changes in length and force capacity.

### Under what conditions are the compliance and activation-dependent shifts in L0 meaningful?

While the analysis of average behavior paints results with a wide brush, it fails to capture the complex interactions between the muscle’s starting passive length, compliance, and activation-dependent shifts in L0. Although there are trends in these interactions, it is important to emphasize that they are not broad. These relationships are very sensitive to a muscle’s passive length and have large continuous gradients that shift with MTU compliance. Thus, small changes in the initial passive length can result in a large change in activation-compliance-dependent force capacity that generalizations fail to capture. For instance, while averages suggest that activation-dependent shifts in L0 have little influence on force capacity, more detailed analysis shows that they can decrease or increase force capacity by as much as 15% of *F*_100max_, resulting in a relative change of over 60% in force capacity at low activations. Likewise the average analysis underrepresented the extent of compliance on muscle length, which can be drastic, resulting in changes of up to 40% of L0, which are commensurate with the largest muscle strains seen in dynamic, high power movements (Askew and Marsh 2002). Here we show this in a fixed-end “isometric” contraction. Further, while the “average” analysis concluded only a small effect of activation on muscle length in non-compliant muscles, this more detailed analysis highlighted that there is no abrupt threshold of MTU compliance below which the effects disappear. Rather there is a non-linear continuous gradient with compliance that varies across muscle operating lengths. There are regions of the F–L curve in which muscles are most sensitive to compliance and there are troughs of influence where these factors have little effect on force capacity. These regions are hard to predict, though, because the magnitude and location of these areas of high and low sensitivity shift with compliance level. Thus, rather than being able to provide an accurate rule of thumb for how and when compliance will alter a muscle’s force generating capacity, we identify conditions in which the effect is most drastic, the variables that are most influential, and provide a guide for the possible errors in predicting muscle function if muscle–tendon properties are not known.

Although, on average, changes in muscle length were greatest for muscles starting at passive lengths closer to optimal fiber length, L0, the influence of compliance on force capacity does not follow suit. The length at which we see the greatest change in force shifts with MTU compliance. On the ascending limb, stiff MTUs show the greatest drop in force at the shortest passive lengths (dark blue regions of Fig. 5D), while compliant muscles see the greatest change when muscles start at passive lengths just short of optimal fiber lengths (yellow regions of Fig. 5D). Even though it is fair to say that compliance will always decrease the force generating capacity of muscles with passive lengths on the ascending limb, the non-linear interactions between activation and compliance make any approximations of the magnitude of the effect potentially inaccurate. Depending on passive muscle length, the influence of compliance could decrease a muscle’s force capacity on the ascending limb anywhere from 0 to 80%. Whereas these numbers are striking, this isn’t even the region of the F–L curve that is most sensitive to the effects of compliance. The region of most variability extends from just short of the plateau region of the F–L curve and down a portion of the descending limb. For muscles within this region, at 100% activation compliance can result in either a decrease or increase in force capacity of well over 50% of *F*_100max_, depending on the passive length. It is important to emphasize that these conditions in which we see the greatest influence of activation on operating length are not unusual. Within this region of highest variability are conditions we typically regard as “optimal,” on or near the plateau. Thus, surprisingly, across the region of the F–L curve we typically view force as relatively insensitive to length changes (i.e., the plateau), we find the area of greatest sensitivity to activation-compliance effects. In summary, compliance results in activation-dependent changes in the operating length and force capacity of muscles, the maximum values of which increase with increasing compliance. On the ascending limb, force capacity decreases with increasing compliance and passive muscle length. On the plateau or descending limb, the story is much more complex and force capacity can either increase or decrease depending on compliance and initial passive muscle length. Thus, at a muscle function level we show that even in “isometric” conditions, the MTU is really “dynamic” with changes in muscle length, force capacity, and activation requirements. One cannot accurately assume force increases proportionally with activation; the activation–force relationship is far from linear. Instead, it is likely a complex interaction of activation, muscle passive length, and MTU compliance. This interaction, although not well understood, suggests great computational complexity for neural control of muscle force production.

The influence of activation-dependent shifts in L0 on force capacity generally follows a similar pattern as described for compliance, increasing with increasing compliance. Again, the effect of compliance can be explained by the dynamic nature of these interactions. The changes in force capacity that come with a shift in L0 result in larger length changes in more compliant MTUs, which result in further force changes. While these effects can be small in magnitude (<5% of maximum force capacity, *F*_100max_ for T/M ratio < 2), at low activation, they can represent a relatively large change in force capacity (over 20%). In fact, at low activation, the influence of activation-dependent shifts in L0 is as important, if not more important, than compliance effects in determining a muscle’s force capacity. Further, there is only a narrow region of muscle lengths that result in “no influence”; thus, the contribution of activation-dependent shifts in L0 is more ubiquitous than our average analysis suggested, substantially altering the force capacity across a wide range of MTU compliance and operating lengths at low activations. In summary, this analysis allows us to provide information on what the most important variables are for accurately predicting muscle force. Compliance is more important than activation-dependent shifts in L0 at high activation, while both significantly alter the force capacity at low activation levels.

The influence of compliance and activation-dependent shifts of L0 has implications for musculoskeletal modeling and can provide a guide for what the possible errors are in predicting muscle function if muscle–tendon properties are not known. Models that assume non-compliant tendons could be over or underestimating muscle force drastically. Specifically, this simplification would most often result in over estimation of force generating capacity with muscles operating on the ascending limb and under estimation on the descending limb. This is true, even within the less compliant range of human MTUs (lower limb av. 3.06 [Arnold and Delp, 2011]). At a T/M ratio of 3, for instance, our analysis shows that for all but a narrow band of passive lengths, muscles show significant force deficits with compliance. More striking is that even muscles with relatively short tendons (tendons shorter than the muscle optimal fiber length, T/M ratios lower than one) have regions of passive lengths on the ascending limb where compliance result in significant changes in force capacity. This implies that even low levels of compliance can significantly alter force capacity in some conditions and that models that ignore or do not accurately define compliance should be interpreted cautiously. More optimistically, muscle models that include compliance but ignore activation-dependent shifts in L0 should capture the most dominant factors when simulating muscles at high activations. But in cases where muscles are at submaximal activation, ignoring activation-dependent shifts in L0 could lead to errors that mirror those of non-compliant models; in general forces on the ascending limb are overestimated and those on the descending limb underestimated.

### What are the functional consequences of compliance/activation-dependent shifts in L0?

While the major focus of this paper is to understand in detail the influence of compliance and activation-dependent shifts in L0 on muscle operating lengths and force capacity, we also make a first pass at assessing their possible functional influence. We do this in a task where our “static” analysis is most applicable, namely in a standing posture.

First, consistent with results from our other analyses, we find that force in the hip muscles, which shorten down the ascending limb, decrease when both compliance and activation-dependent shifts in L0 are included in the muscle models, whereas forces in the ankle muscles, which shorten up the descending limb, increase with the addition of activation-dependent effects. These results are also consistent with our finding that activation-dependent effects generally have a larger influence on more compliant MTUs. Models that include both activation-dependent effects have a greater influence on “optimal force-generating posture” at the ankle than at the hip. Surprisingly, though, the influence of activation-dependent shifts in L0 are greater for muscles acting at the hip than those acting at the ankle, despite our results which show that activation-dependent shifts generally have larger effects for more compliant muscles. This emphasizes two take home points from this study. 1) Muscles with very little tendon are not immune to non-linear activation-dependent effects, and 2) as stated previously, the magnitude of activation-dependent effects is very sensitive to the lengths at which the muscles operate. Here, the muscles acting across the ankle fall into a trough of influence in the plateau region where activation-dependent shifts in L0 have little impact (red star, Fig. 6C), while the muscles acting at the hip operate on the ascending limb in a region of relatively high influence (gray star Fig. 6C). Thus, again, we emphasize how highly sensitive activation-dependent effects are to muscle passive lengths and how broad generalizations can lead to erroneous conclusions.

In a previous study, we also asked whether animals utilize postures that maximize torque capacity during walking and/or running. These analyses concluded that they did not, but were based on simpler muscle models that did not incorporate activation-dependent effects (compliance or activation-dependent shifts in L0 [Hutchinson et al. 2015]). This previous study also did not separate the roles of length-dependent muscle force capacity and muscle moment arms in dictating the effect of joint posture on moment capacity. Several experiments have shown that peak isometric muscle force and moment arms do not necessarily coincide with the joint angle that generates peak muscle or joint moments (Lieber and Boakes 1988a; Hoy et al. 1990). Whether muscle force or moment arms have the largest contribution to peak moment is variable. For example, for the same muscle (bi-articular frog semitendinosus), force capacity dictates the muscle’s peak knee joint moment (Lieber and Boakes 1988b), but the muscle’s moment arm dictates the moment-angle profile at the hip (Lieber and Shoemaker 1992). How length-dependent force capacity and muscle moment arms influence observed postures remains poorly understood.

If we assume that models that incorporate both compliance and activation-dependent shifts in L0 accurately capture muscle dynamics in this species and a conservative 25% muscle activation, we do find some evidence for muscle–tendon mechanics and posture operating in concert. Notably, we find that at the ankle, animals stand with a posture that maximize both force and joint moment, accommodating the strain in the muscle that occurs due to the high compliance of its MTUs. These could offer support to the long-held theory that muscles operate at a length that minimize force losses arising from their F–L relationship (Smith et al. 2007; Azizi and Roberts 2010; Lieber and Ward 2011; Rubenson et al. 2012; Azizi 2014).

However, the story is complex. At the hip, we see a difference of ∼45° between postures that maximize torque and force, with the animal choosing a posture halfway between. Standing posture is ∼22° more extended than optimal for force production and ∼22° more flexed than the posture that maximize joint torque. This symmetry obscures the functional significance of these differences, though. Since force capacity is less sensitive to changes in joint angle, a postural difference of 22° results in a loss of only 20% in force capacity but decreases joint moment by nearly 40%. It is interesting to note that adopting a posture that would increase moment capacity at the hip (more erect) would also decrease the moment necessary to oppose gravity. Thus, a less flexed hip angle could be more economically supported, yet is not adopted. While 25% activation may overestimate muscle activations during standing (Rubenson and Marsh 2009), a lower activation level will only amplify these effects. Thus, this study, along with our previous work and those of others, hints that there are likely many factors that influence preferred posture and that torque capacity is not universally prioritized. While our results are among the first to systematically link length-dependent force capacity with posture, whether this specific mechanical constraint strongly dictates posture is still far from clear.

### Limitations and future directions

While we have done these analyses assuming a particular level of activation-dependent shift of the F–L curve, several studies have suggested that the level of shift may vary by species and may range from having no influence (de Brito Fontana and Herzog 2016), to as large as 60% shift (Holt and Azizi 2014). Our analysis of a 15% shift level was not intended to produce a definitive quantification of its influence, but as a first pass at evaluating the possible contribution and interaction effects. These analyses could be repeated for different levels in the activation-dependent shift in L0 and in dynamic conditions that include force–velocity effects to quantify how this influences the general conclusions. Likewise, care should be taken in extrapolating our results for species with different tendon properties since variations in tendon stiffness could result in very different force and length changes for the same ratio of muscle to tendon length. Regardless of these limitations, this analysis highlights the advantages of exploring these questions via musculoskeletal modeling, an approach which allows the generation of thousands of data points to elucidate patterns and trends that are not visible from studies of one or two muscles alone.

## Data availability

Model and data can be found at https://simtk.org/projects/guineafowl.

## Author contributions

S.M.C. and J.R. contributed to the conception and design of the study, drafted the initial manuscript, and contributed to the figure preparation. S.M.C., K.L.E., S.L.D., and J.R. were the primary authors involved in the development of the musculoskeletal model and S.M.C developed the muscle simulations. M.C.L., R.L.M., and J.R. were the primary authors involved in the collection and analysis of experimental data. All authors contributed critically to the data interpretation. S.M.C., K.L.E., R.L.M., S.L.D., and J.R. contributed to editing the manuscript.

## Supplementary Material

obz022_Supplementary_Data
